# Comparing time-series transcriptomes between chilling-resistant and -susceptible rice reveals potential transcription factors responding to chilling stress

**DOI:** 10.3389/fpls.2024.1451403

**Published:** 2024-08-06

**Authors:** Rui Zhang, XiaoHui Xi, XinYi Chen, Yi Wang, Ming Zhou

**Affiliations:** ^1^ State Key Laboratory of Plant Environmental Resilience, College of Life Sciences, Zhejiang University, Hangzhou, China; ^2^ Key Laboratory of Nuclear Agricultural Sciences of Ministry of Agriculture and Zhejiang Province, Zhejiang University, Hangzhou, China

**Keywords:** comparative transcriptome, gene regulatory network, transcription factors, chilling stress, rice

## Abstract

Low temperature is one of the most important environmental factors that inhibits rice growth and grain yield. Transcription factors (TFs) play crucial roles in chilling acclimation by regulating gene expression. However, transcriptional dynamics and key regulators responding to low temperature remain largely unclear in rice. In this study, a transcriptome-based comparative analysis was performed to explore genome-wide gene expression profiles between a chilling-resistant cultivar DC90 and a chilling-susceptible cultivar 9311 at a series of time points under low temperature treatment and recovery condition. A total of 3,590 differentially expressed genes (DEGs) between two cultivars were determined and divided into 12 co-expression modules. Meanwhile, several biological processes participating in the chilling response such as abscisic acid (ABA) responses, water deprivation, protein metabolic processes, and transcription regulator activities were revealed. Through weighted gene co-expression network analysis (WGCNA), 15 hub TFs involved in chilling conditions were identified. Further, we used the gene regulatory network (GRN) to evaluate the top 50 TFs, which might have potential roles responding to chilling stress. Finally, five TFs, including a C-repeat binding factor (*OsCBF3*), a zinc finger-homeodomain protein (*OsZHD8*), a tandem zinc finger protein (*OsTZF1*), carbon starved anther (*CSA*), and indeterminate gametophyte1 (*OsIG1*) were identified as crucial candidates responsible for chilling resistance in rice. This study deepens our understanding in the gene regulation networks of chilling stress in rice and offers potential gene resources for breeding climate-resilient crops.

## 1 Introduction

Higher plants encounter a wide variety of environmental stresses in their growth and development (Nawaz et al., 2023). The chilling stress (0°C–15°C) is one of main abiotic stresses bringing serious negative impacts on plant development and causing huge economic losses in crops (Ding et al., 2019). Encountering with low temperatures, crops, especially in the tropical and subtropical climatic zones, show irreversible physiological and biochemical perturbations, including leaf falling, incomplete ripening, seedling wilting, and even death (Lukatkin, 2005; Xu et al., 2008; Lukatkin et al., 2012; Sun et al., 2017).

To gain deeper insights into gene networks responding to the chilling stress, plenty of differentially expressed genes (DEGs), especially for transcription factors (TFs), have been identified when treated with chilling stress (Seki et al., 2001). These TFs play key roles in responding to chilling in plants. For example, overexpression of the *C-repeat binding factor1* (*CBF1*) triggered the expression of cold-responsive (COR) genes and, subsequently, the identification of ICE (inducer of *CBF* expression) function led to the establishment of the ICE-CBF-COR pathway to enhance the chilling resistance in *Arabidopsis* (Thomashow, 1999; Chinnusamy et al., 2006; Nie et al., 2022). Overexpression each of *osmotic stress/abscisic acid (ABA)–activated protein kinase 6* (*OsSAPK6*), *ideal plant architecture 1* (*IPA1*), and *OsCBF3* forms a chilling-induced OsSAPK6-IPA1-OsCBF3 signal cascade, and enhances rice chilling tolerance (Jia et al., 2022). Other TFs, such as *MaNAC1*, *OsWRKY63*, and *ZmMYB31*, are also involved in chilling tolerance by regulating the expression of downstream genes as well (Li et al., 2019; Zhang et al., 2022; Yin et al., 2024). In addition, plant hormones usually show a close connection with chilling stresses. ABA regulates several biochemical functions including fruit ripening, leaf senescence, and stomatal closure (Kumar et al., 2022). Studies also show that chilling stress impacts the development and stress resistance of plants by influencing the accumulation of endogenous ABA or inducing key molecular hub genes in ABA synthesis and transduction pathways (Guan et al., 2023). In tomato, overexpression of *abscisic acid-insensitive 5* (*MaABI5-like*) increases fatty acid desaturation and enhances cold tolerance (Song et al., 2022). In melon seedlings, *abscisic acid-responsive element-binding factor 1* (*CmABF1*) and *CmCBF4* cooperatively regulate putrescine synthesis to help melon seedlings to response to chilling stress (Li et al., 2022).

Rice, which is cultivated across an expanse of 164 million hectares in over 100 countries, serves as a major staple food crop and provides dietary energy for half of the population in the world (Shafi et al., 2023). Since it is a tropical and subtropical crop, the growth of rice is sensitive to the chilling stress, which affects the seedling growth, heading, grain filling, and seed setting of rice. Therefore, understanding gene networks responsive to the chilling stress will provide crucial clues for breeding climate resilient rice breeding. DC90 is a chromosome segment substitution line that is introgressed with the chilling-tolerance locus CTS-12 from the wild rice, while 9311 is a chilling-sensitive cultivar. After 5 days chilling treatment, there was no significant difference between DC90 and 9311, and phenotypic differences were observed to develop gradually between DC90 and 9311 during the recovery period. After 7 days recovery, the seedlings of 9311 were completely wilted, and the seedlings of DC90 were survived after the chilling stress (Cen et al., 2018, Cen et al., 2020). In previous study, DEGs in DC90 and 9311 under 72h chilling treatment (time-course RNA-seq at 0h, 12h, 24h, and 72h) and 24h recovery (at 84h and 96h) have been characterized by Cen et al (Cen et al., 2020). Nevertheless, transcriptional dynamics and crucial TFs responding to low temperature between DC90 and 9311 at various time points remain unclear. Thus, we aimed to conduct weighted gene co-expression network analysis (WGCNA) and gene regulatory network (GRN) analyses and identified potential TFs responsible for chilling resistance in rice.

In this study, we re-analyzed time-course transcriptomes from DC90 and 9311. The expression data were compared at 0h, 12h, 24h, 72h, 84h, and 96h between two rice cultivars through co-expression and regulatory networks. We revealed key genes that had positive and negative regulatory effects on the low temperature and constructed a gene co-expression network for chilling stress in rice. Further, we discovered that crucial genes involved in the ABA synthesis and signal-transduction pathway that were induced by low temperature, suggesting ABA might plays a significant role in chilling signaling cascades. Importantly, several TFs potentially responsible for chilling resistance and their relationship with each other in the regulatory network were identified. Collectively, our findings identified a hub-TFs regulatory network of chilling stress in rice. These data might provide clues for breeding climate-resilient crops.

## 2 Materials and methods

### 2.1 Plant materials and data sources

DC90 and 9311 are two rice cultivars that are resistant and sensitive to the chilling stress, respectively (Cen et al., 2018, Cen et al., 2020). All rice seedlings (three-leaf stage) were grown in Guangxi University, China, and then exposed to low temperature at 10°C/8°C (day/night) (artificial light was supplemented with day-13h/night-11h circadian rhythm). The seedling leaves were collected at 12h, 24h, and 72h under chilling stress treatments, and at 84h and 96h for recovery stages; 0h was set as control. The collected samples were immediately ground with liquid nitrogen with three biological replicates. The paired-end reads were generated from Illumina 2500 platform and the RNA-seq datasets were deposited in NCBI under the accession number PRJNA601963.

### 2.2 Differential gene expression analysis

For RNA-seq data, we employed Fastqc v0.11.9 (https://www.bioinformatics.babraham.ac.uk/projects/fastqc/) to examine sequence quality and Trimmomatic v0.39 (Bolger et al., 2014) to remove low-quality reads. The clean reads were mapped to rice msu7 genome (http://rice.uga.edu/downloads_gad.shtml) with Hisat2 v2.2.1 (Kim et al., 2019). Gene expression was uniform in fragments per kilobase of exon model per million (FPKM) mapped reads using feature Counts v2.0.1 (Liao et al., 2014). DEGs at 6 time points related to chilling stress between DC90 and 9311 were calculated using the R package DESeq2 (Love et al., 2014). The threshold of DEGs was set to |log_2_ Fold Change| ≥ 1 and p.adjust ≤ 0.05. These DEGs were grouped as upregulated or downregulated genes based on positive or negative logarithmic changes in threshold value. Heatmaps and Venn diagrams were drawn with the R packages ggplot2 (Ginestet, 2011) and UpSetR (Conway et al., 2017).

### 2.3 Co-expression network recognition based on the expression level of DEGs

Co-expression network analysis was performed using the R package WGCNA (Langfelder and Horvath, 2008). Briefly, the FPKM matrix of all DEGs was used as input data and these datasets of all time points with three replicates were filtered to remove some genes at low-expression level (FPKM <1) of any sampling replicates. For these resulting genes, we firstly estimated the soft threshold power by the pickSoftThreshold function, which provided the best-weighted coefficient β = 8 for the scale-free network construction. Second, a correlation matrix was constructed and a topological overlap matrix was calculated based on this correlation matrix. Finally, these genes were classified into different modules according to the topological overlap dissimilarity by average hierarchical clustering algorithm. These modules with different colors were then calculated based on a dynamic tree with minimum group size of 30 genes and merging threshold function of 0.25. Hub genes can represent the maximum correlation points with each module and they are usually represented by the kME value. The threshold used to screen hub genes in each module was |kME| > 0.85. The intricate networks were achieved based on the kME value by employing Cytoscape v3.8.2 (Gnanaraj, 2008).

### 2.4 Gene ontology annotation and enrichment analysis

Gene Ontology (GO) annotation was conducted using the Ensembl (Hubbard et al., 2002). Functional enrichment analysis was exhibited by the R package clusterProfiler v4.6.2 (Wu et al., 2021) with p.adjust ≤ 0.05. All dotplots were visualized with the R package ggplot2 (Ginestet, 2011).

### 2.5 Construction of GRN

According to the PlantTFDB v5.0 (https://planttfdb.gao-lab.org/download.php) annotations, 261 of 3,590 genes encoded TFs were identified. These 261 genes were defined as potential regulators based on the machine learning. We used the R packages GENIE3 (Vân and Geurts, 2018) to measure the confidence levels (“weights” ≥ 0) and the top 10,000 inferred regulatory relationships were found from GRN as potential GRN. The top 50 TFs with the heaviest summed weights in the GRN were retained as the candidates. The networks were drawn by Cytoscape v3.8.2 software (Gnanaraj, 2008).

## 3 Results

### 3.1 Identification of DEGs under chilling stress between two rice cultivars

**Figure 1:**
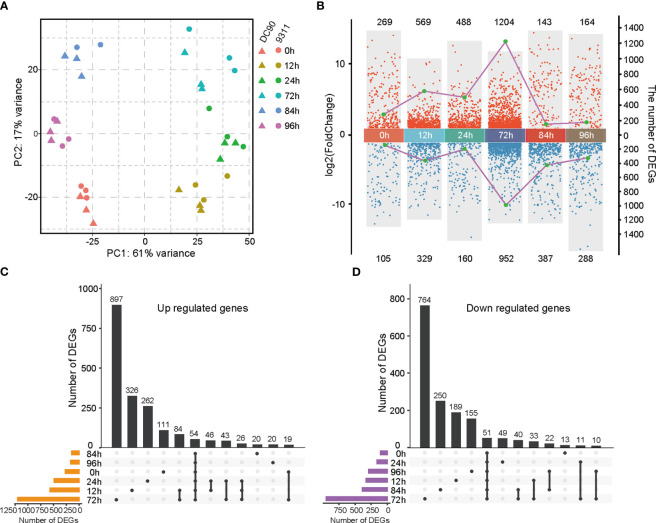
Transcriptomic analyses of DC90 and 9311 under 72h chilling treatment and 24h recovery. **(A)** Principal component analysis (PCA) of DC90 and 9311 samples at 6 time points (0h, 12h, 24h, 72h, 84h, and 96h). **(B)** Statistics of differentially expressed genes (DEGs) numbers in DC90 relative to 9311 at 6 time points. Each dot presents a DEG. Dots in blue show downregulated genes, and dots in red represent upregulated genes. The left coordinate (y-axis) represents the log_2_ fold change values of DEGs and the right coordinate (y-axis) represents the numbers of DEGs. **(C)** Upset plot shows unique and shared upregulated genes at each time point in DC90 relative to 9311. **(D)** Upset plot shows unique and shared downregulated genes in DC90 relative to 9311.

DC90 and 9311 are two rice cultivars that are resistant and sensitive to the chilling stress at seedling stage, respectively. To understand their distinguished responses to chilling stress at transcriptional level, samples were collected for time-course transcriptomes (Cen et al., 2020). For the chilling treatment (see Methods), samples were collected at 0h, 12h, 24h, and 72h, while samples were subsequently collected at 84h and 96h for recovery (Cen et al., 2020). To explore the transcriptome dynamics responding to chilling stress in DC90 and 9311, we analyzed the complete gene expression in seedlings at the 6 time points with three biological replicates. Based on principal component analysis, we found that all time points in two rice cultivars could be divided into two main components (**Figure 1A**). The first principal component (PC1) accounted for 61% of the variance and was explained by samples with chilling stress and samples without treatment or after recovery. The second principal component (PC2) accounts for 16% of the variation and was interpreted by the genetic differences between DC90 and 9311. This result indicates that transcriptomes of the chilling stress do not only reflect the chilling treatment versus the recovery condition but also present a unique transcriptome composition in different genetic backgrounds.

To identify DEGs responding to chilling stresses, pairwise comparisons among the same 6 points of DC90 relative to 9311 were performed using the DESeq2 program (Love et al., 2014). A total of 3,590 genes were obtained showing a clear distinction between different treatment points and the detailed information for those DEGs were listed in **Supplementary Table S1**. Among these 6 time points, the greatest number of DEGs was found after 72h chilling treatment (1,204 upregulated and 952 downregulated) (**Figure 1B**). Most of these DEGs were unique at 72h, including 897 unique upregulated genes and 764 unique downregulated genes (**Figures 1C, D**), indicating that a longer duration of exposure to the chilling stress led to profound changes of rice transcriptome profile. According to the multiple-point comparison, we detected 54 upregulated DEGs and 51 downregulated DEGs that commonly changed at 6 time points between DC90 and 9311 (**Supplementary Tables S2**, **S3**). Globally, the number of upregulated DEGs was more than the number of downregulated genes under chilling treatment (0h, 12h, 24h, and 72h), while the number of downregulated DEGs was more than the number of upregulated genes at each time point under the chilling recovery stages (84h and 96h). These results presented a huge difference of DEGs in response to chilling stress between these two rice cultivars.

### 3.2 Co-expression analysis reveals co-regulated modules of chilling responsive genes

**Figure 2:**
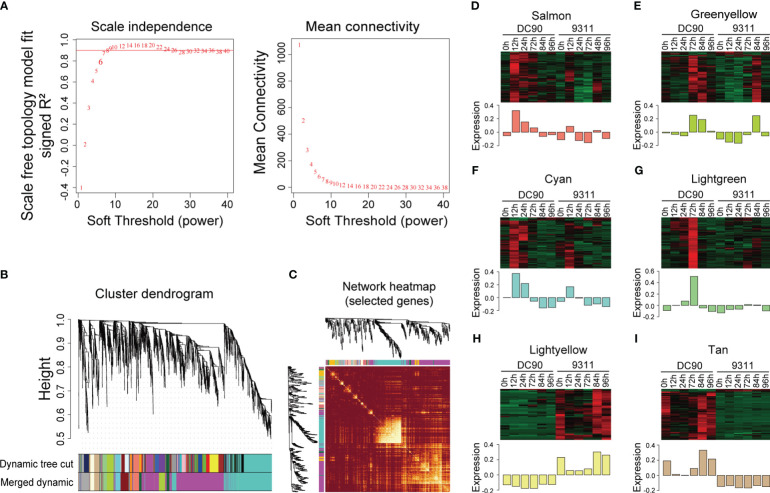
Weighted gene co-expression analysis (WGCNA) of DEGs between DC90 and 9311 under 72h chilling treatment and 24h recovery. **(A)** Scale independence and mean connectivity of the network for various soft-thresholding powers. **(B)** A hierarchical cluster tree of the DEGs distributed in different modules. The assigned modules are depicted by color bands and branches. Genes are represented by the tips of the branches. **(C)** The correlation between co-expression networks based on eigengenes. The intensity of color on the heatmap signifies the extent of overlap and the darker yellow denoting greater correlation. **(D–I)** Expression patterns of co-expressed genes in six modules, including **(D)** salmon, **(E)** green yellow, **(F)** cyan, **(G)** light green, **(H)** light yellow, and **(I)** tan modules. The expression quantity was processed with z-score normalization, and the relative expression levels are shown in colorful scale.

In order to investigate key regulators responsive to chilling stress in rice, lowly expressed gene (the FPKM value <1) were filtered, and remaining DEGs were further employed for WGCNA analysis (Langfelder and Horvath, 2008). We constructed the analysis of network topology for soft-thresholding powers from 1 to 40 and identified a relatively balanced scale independence and mean connectivity (**Figure 2A**). In the current research, β = 8 was selected with scale free (*R* ^2^ = 0.9030) to ensure the network topology. Then a gene tree based on hierarchical cluster were established, and a total of 12 co-expression modules with were obtained except the gray module which was contained the genes (*n* = 524) that were not effectively clustered (**Figure 2B**). All genes in the same module shared the similar expression patterns and the number of DEGs in these modules ranged from 35 DEGs (the dark orange module) to 1,291 (the magenta module) (**Supplementary Table S4**). We identified eigengenes (ME, defined as the first principal component of genes in module) as representative patterns of module and evaluated the similarity of each module by correlating their eigengenes (**Figure 2C**).

According to these eigengenes expression patterns, we found that at 3 time points after chilling stress, expression levels of eigengenes including salmon, green yellow, cyan, and light green modules were showed significantly higher in DC90 when compared to 9311. At the same time, expression levels of DEGs in these module eigengenes were higher under chilling stage (12h, 24h, and 72h) compared to unchilling stage (0h, 84h, and 96h), indicating that they were consistently stimulated in DC90 and might play crucial roles in response to chilling stress (**Figures 2D–G**). In addition, some modules also correlated with specific genotypes according to expression profiles of eigengenes (**Figures 2H, I**). The eigengenes of DC90 showed higher expression levels in tan modules but showed lower expression levels in light yellow modules when compared to 9311, suggesting that differences in gene expression between these two modules are mainly due to variety differences, rather than being significantly correlated with cold stress.

### 3.3 Gene ontology enrichment analysis of DEGs in different modules

**Figure 3:**
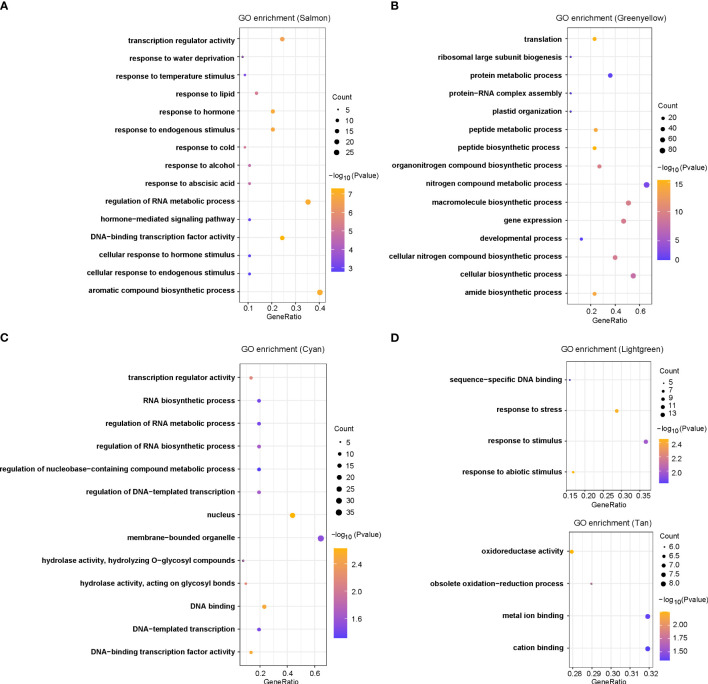
GO enrichments for co-expression genes in five selected modules, including **(A)** salmon, **(B)** green yellow, **(C)** cyan, **(D)** light green, and **(E)** tan modules.

To assess if these DEGs have any biological function in response to chilling stresses, we used gene ontology functional enrichment analyses for DEGs corresponding to each module and the top 15 GO terms in each module were given (**Figure 3A**; **Supplementary Figure S1**). For the six modules in **Figures 2D–I**, we have discovered many terms potentially involved in chilling stress response. In the salmon module, the most enriched significant processes contained transcription regulator activity, temperature stimulus, cold, hormones, hormone-mediated signaling pathways and cellular responses to hormone stimulus (**Figure 3A**). These results suggested that plant hormones might play key roles in response to chilling stress in rice. In the green yellow module, the most enriched significant processes were responsive to translation, peptide biosynthetic process, peptide metabolic process, and protein metabolic process, suggesting that genes in this module were likely involved in protein synthesis and metabolism (**Figure 3B**). In the cyan module, transcription regulator activity was the most significant function-enriched GO term, indicating that the TFs might play crucial roles in response to chilling stress (**Figure 3C**). In the light green module, the most significant processes were enriched for sequence-specific DNA binding, response to stress, and response to stimulus, while in the tan module, oxidoreductase activity, metal ion binding, and cation binding were enriched (**Figures 3D, E**). Although the genes in light yellow modules were not significantly enriched in any GO functions, we found that the GO annotations of many genes in this module were related to calmodulin-binding protein, oxidoreductase, and heavy metal-associated (**Supplementary Table S4**). Taken together, the analysis reveals that chilling stress influences the expression of genes that regulate plant hormone levels, protein contents, and TF activities, and these DEGs may be severed as a versatile resource for breeding chilling-resistant plants, thus we keep salmon, green yellow, cyan, and light green modules as the “rice chilling resistance response” modules for subsequent analyses.

### 3.4 ABA biosynthesis and signaling genes actively respond to the chilling stress

**Figure 4:**
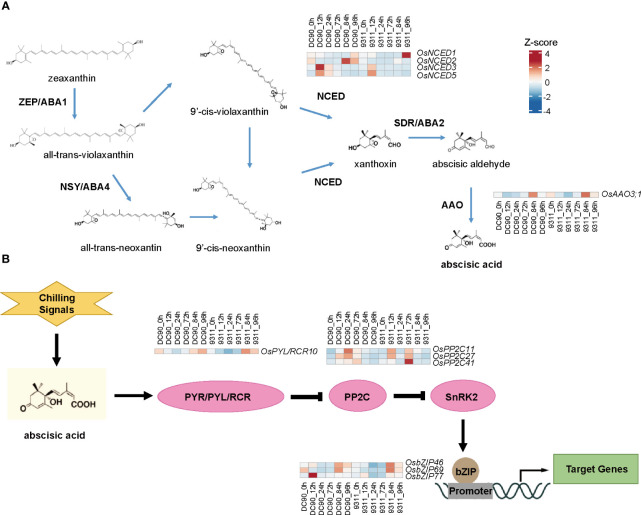
Heatmaps of related differentially expressed genes (DEGs) in abscisic acid (ABA) biosynthesis and signaling pathways. **(A)** Heatmaps of relevant genes involved in the pathway of ABA biosynthesis. **(B)** Heatmaps of relevant genes involved in the pathway of ABA signaling. The heatmap indicates z-score normalized expression levels of DEGs. The red color presents upregulation of genes and blue indicates downregulation of genes.

In the GO analysis, we found that ABA was a highly enriched term, which is consistent with previous study (Cen et al., 2020). To understand roles of ABA in rice chilling stresses, the intricate synthesis and signal-transduction pathway of ABA responding to low temperature were further analyzed (**Figure 4**). In the ABA biosynthesis pathway, *9-cis-epoxycarotenoid dioxygenase* (*OsNCED3/5*) displayed a high expression level in DC90 than that in 9311 after 12h chilling treatment, whereas *aldehyde oxidase* (*OsAAO3;1*) showed a low expression level at all chilling hours in two rice cultivars (**Figure 4A**). Of particular significance, genes situated at the inception of the ABA signal-transduction cascade exhibited obvious induction in the wake of chilling signals. (**Figure 4B**). Among them, *OsPP2C11*, *OsPP2C27*, and *OsPP2C41* showed high expression profiles in 9311 at 72h than that in DC90 that might because of PP2Cs as a negative regulator of ABA signaling pathway. We also investigated expression levels of genes involved in other hormone pathways, such as brassinosteroids (BR), cytokinin (CTK), ethylene (ETH), gibberellin (GA), auxin (IAA), and jasmonic acid (JA), but no significant differences were found between these two varieties under chilling stress (**Supplementary Figures S2**–**S7**). These results indicate that under low temperature stress, ABA synthesis and signal transduction pathways play key roles in responding to chilling stress in DC90 versus 9311.

### 3.5 Identification of hub TFs responding to the chilling stress

**Figure 5:**
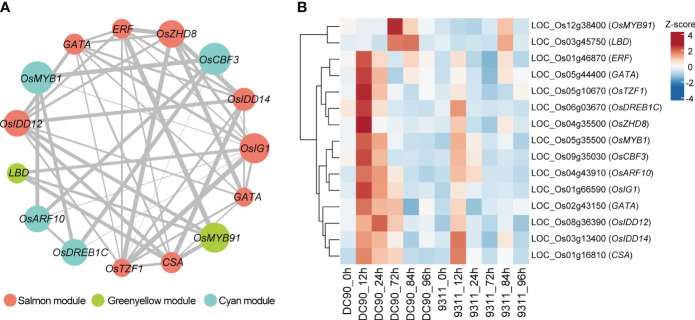
Identification of hub transcription factors (TFs) in three selected modules involved in chilling stress and their co-expression network. **(A)** Visualization of the 15 key TFs co-expression networks. Hub TFs of salmon, green yellow, and cyan modules are indicated with salmon, green yellow, and cyan color, respectively. Gray lines indicate the correlations between hub TFs. The size of the points represents the level of |KME|, and the width of the gray line represents the strength of co-expression intensity. **(B)** The heatmap of these 15 hub TFs in DC90 and 9311. The values in the heatmap represent the z-scores of FPKM at 6 time points in two cultivars. The red and blue colors indicate a high and low expression profiles, respectively.

TFs served as key regulators to regulate plant development and responses toward the chilling stress (Kaufmann et al., 2010). To investigate hub TFs and genes involved in chilling resistance response, we calculated the module membership values (kME) among the four “rice chilling resistance response modules” based on WGCNA packages. We chose candidate TFs with the |kME| >0.85 in these modules, and this analysis retained a lot of hub genes in each module (**Supplementary Table S5**). Based on WGCNA analysis, totally, we identified 15 hub TFs from four modules that belong to MYB, ethylene-responsive element binding factor (ERF), zinc finger and INDETERMINATE DOMAIN protein (IDD), lateral organ boundaries domain (LBD), and other TF families (**Figure 5A**). These TFs formed a complex regulatory network, which may play crucial roles in chilling resistance in rice. We also investigated the expression profiles of these 15 TFs in DC90 and 9311 among six different points (**Figure 5B**). We found 13 of these 15 TFs were highly induced at 12h or 24h after chilling stress in DC90 compared with that in 9311, while the other two TFs were highly accumulated after 72h chilling stress in DC90. All these 15 TFs expressed higher under chilling stress (12h, 24h, and 72h) in DC90 than that of 9311, suggesting that these TF might serve as key TFs to regulate the chilling resistance.

### 3.6 Inferring the TFs regulatory network by machine learning

WGCNA is an effective method to explore the gene co-expression networks and hub genes; however, the regulatory relationships between different genes are not easy to evaluate through this method. An analytical pipeline (see Methods) suggested 15 TFs served as reliable candidates for the chilling response network (**Figure 5**), but the regulation direction between these TFs was not captured. Therefore, we employed the GENIE3 package (Vân and Geurts, 2018), which was used to predict the regulatory relationships of different genes and TFs based on a machine learning algorithm.

**Figure 6:**
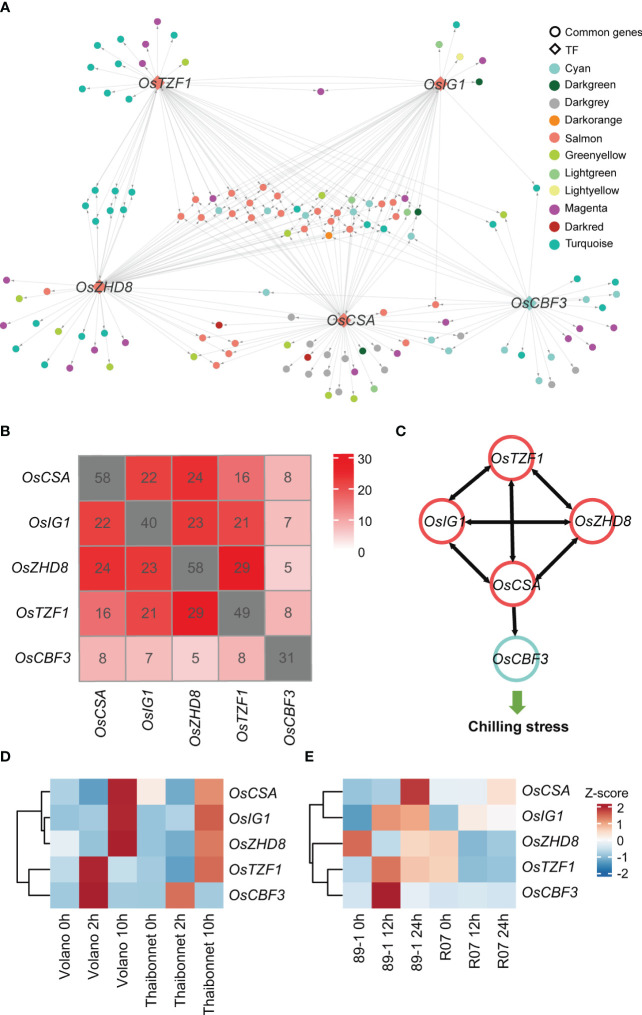
Identification of crucial transcription factors (TFs) and their target genes in a gene regulatory network and temporal expression profiles of five key TFs in different cultivars under chilling stress. **(A)** An overview of the transcriptional regulatory sub-network centered by five TFs. Each point represents a gene. Genes with regulatory relationships are linked with lines. The direction of gene regulation is indicated by gray arrow. TFs are represented by diamond frames, while other genes are represented by circles. Genes from different co-expression modules are shown in different colors, consistent with the colors in **Figure 2**. **(B)** Overlaps among the predicted target genes of five TF candidates in GRN. The numbers of predicted TF target genes are marked in gray squares. **(C)** Illustration of mutual regulation relationships among the five TFs. Gene regulation directions are indicated by black arrows. TFs from the salmon and cyan modules are marked using salmon and cyan circles, respectively. **(D)** The expression levels of five key TFs in other independent chilling-resistant Volano and chilling-susceptible Thaibonnet varieties of rice under 4°C for 0h, 2h, and 10h. **(E)** The expression levels of five key TFs in the chilling tolerance glutinous rice 89-1 and the susceptible indica rice R07. The red and blue colors indicate high- and low-expression levels, respectively.

According to the random forest method, 50 TFs, which rapidly respond to the chilling stress, with the potential regulatory relationships were explored by GENIE3 (**Supplementary Table S6**). After associating these 50 TFs with 15 hub TFs presented in former modules (salmon, green yellow, cyan, and light green) related to chilling response, we finally retained five TFs, *OsCBF3* (LOC_Os09g35030, an ERF family member), *OsZHD8* (LOC_Os04g35500, a ZF-HD family member), *OsTZF1* (LOC_Os05g10670, a C3H family member), *OsCSA* (LOC_Os01g16810, an MYB family member), and *OsIG1* (LOC_Os01g66590, an LBD family member), as key regulators controlling gene expression networks responding to chilling stress in rice (**Figure 6A**; **Supplementary Table S7**). Among these TFs, OsCBF3 is a well-studied TF in rice chilling stress. In rice, OsSAPK6 interacts with IPA1 and phosphorylates IPA1 to stabilize it, allowing IPA1 to accumulate and activate the expression of OsCBF3 downstream to enhance rice chilling stress resistance (Jia et al., 2022). Thus, this analysis indicates our analysis is efficient and reliable.

Subsequently, we extracted the sub-regulatory network based on these five TFs of the entire gene regulator network (GRN). This subset of data contained 141 genes for salmon, greenyellow, cyan, and lightgreen modules accounting for approximately 59.75% of the total 236 genes of the entire sub-network (**Figure 6A**). A lot of genes were simultaneously regulated by multiple TFs (**Figure 6B**). For example, *OsCBF3* in cyan module was calculated to be regulated by *OsCSA*, and the other four TFs (*OsTZF1*, *OsZHD8*, *OsIG1*, and *OsCSA*) in the salmon module were also predicted to regulate each other, indicating a complex feedback regulation network among the crucial regulators (**Figure 6C**).

In our analysis, we uncovered that five TFs might play as key regulators that fitness the resistance of chilling stress in DC90 compared to 9311. To validate the accuracy of our results, we used transcriptomes derived from the chilling resistant (Volano) and chilling susceptible (Thaibonnet) varieties treated with chilling stresses (4°C for 0h, 2h, and 10h) conditions (**Figure 6D**). We found that *OsTZF1* and *OsCBF3* were highly expressed at 2h, while *OsCSA*, *OsIG1*, and *OsZHD8* were showed higher expression at 12h in the chilling resistant cultivar Volano than Thaibonnet. Similar result was also detected in the tolerance glutinous rice 89-1 and the susceptible indica rice R07 (**Figure 6E**). Taken together, we suppose that these five TFs serve as major and common TFs responding to the chilling stress and play crucial roles in rice chilling resistance.

## 4 Discussion

Chilling stress often leads to a severe decline for rice growth distribution. Therefore, exploring new rice chilling tolerance regulatory genes/TFs will provide key clues for breeding climate-resilient rice. To this end, understanding the transcriptome dynamics under chilling stress in rice is crucial. In our study, we investigated a set of time-course transcriptomes in rice of DC90 (a chilling-resistant cultivar) and 9311 (a chilling-susceptible cultivar) treated with a low-temperature stress. We revealed 12 modules based on the DEGs and a set of hub genes/TFs involved in chilling stress.

Chilling stress can induce alterations of the gene expression profiles in plants. We have found that a lot of DEGs showed rapidly changes at transcriptional levels in response to chilling stimuli in DC90 and 9311. However, it is difficult to explore crucial genes, which might involve in the chilling conditions because of numerous members of DEGs. WGCNA was confirmed as an effective method to help researchers find key hub genes related to specific traits among a large concentration of DEGs (Kuang et al., 2021; Chen et al., 2024; Hu et al., 2024; Xie et al., 2024). In this research, we have divided the DEGs involved in chilling stress into 12 co-expression modules using the WGCNA algorithm to find key genes. Performing functional enrichment annotation on these modules, we found some significant GO terms of “response to temperature stimulus,” “response to hormone,” “protein metabolic process,” and “transcription regulator activity.” These results revealed complicated networks in rice seedlings responsible for or adaptation to the chilling stress.

Based on WGCNA, our analysis revealed some “rice chilling resistance response” modules (salmon, green yellow, cyan, and light green modules), and 15 hub TFs from these modules involved in chilling conditions in rice. Among these 15 TFs, some of them have been validated and confirmed as key TFs related to the chilling stress and other abiotic stress, such as *OsDREB1C*, *OsIDD14*, *OsIDD12*, and *OsMYB91*. *OsDREB1C* serves as a DREB homolog encoding an SLK protein which has been proved to response to chilling acclimation (Mao and Chen, 2012). IDD14 protein interacts with IDD12 and IDD13 to form a transcriptional complex, activating MAPK expression (Cui et al., 2022). *OsMAPK3* and *OsMAPK6* positively regulate rice chilling tolerance and *OsMAPK3* can effectively respond to ABA stimulation, so we speculate that *IDD12/14* might response to low temperature through MAPK cascade signaling pathway (Liu et al., 2023). In addition, overexpression of *OsMYB91* enhances tolerance to abiotic stress and alters the sensitivity of seed germination to ABA (Zhu et al., 2015). Thus, our data support that these 15 TFs are potential regulators in chilling stress response. However, it is noted that roles of these TFs in low temperature remain to be further validated.

Plant hormones, such as BR, ETH, JA, and ABA, have been reported to play key roles in the chilling stress (He et al., 1990; Cao et al., 2012; Xiang et al., 2017; Shu et al., 2022). We found ABA was a crucial factor in response to chilling stress in DC90 versus 9311. Previous study showed that ABA response genes were regulated by *OsCBF3* to resist low temperature (Jia et al., 2022; Li et al., 2022). The chilling stress dramatically increases endogenous ABA level in rice, which results in a significant upregulation of genes involved in ABA biosynthesis and signaling pathways (Xiang et al., 2017). The silencing of *ABI5*, which is composed of PYR/PYL/RCAR receptors in the core ABA signaling pathway, significantly reduces the cold resistance in both rice and banana (Song et al., 2022). *OsNAC5* stabilizes the *OsABI5* protein by inhibiting proteasome-mediated degradation to improve the cold resistance of rice (Li et al., 2024). In our data, the ABA pathway genes *OsNCED1*, *OsPP2C2/5/7*, and *OsbZIP77* showed higher expression levels at 12h under low temperature in DC90 than in 9311. Although *OsPYL/RCR10*, *OsAAO3;1*, and *OsbZIP46/69* all showed lower expression profiles in these two species under chilling stimulate, they were upregulated at 84h (recovery stage), suggesting that they might be some negative regulatory factors involved in chilling stress.

Although WGCNA is an effective method for analyzing gene co-expression networks, the regulatory relationships between co-expressing genes need to be explored. Here, we used a machine learning algorithm GENIE3, which was based on the inference of a collection of regression trees to predict regulatory networks. In previous studies, many nitrogen deficiency-responsive genes and TFs were predicted to function as key regulators within the network in response to nitrogen deficiency based on GENIE3 in rice (Ueda et al., 2020). In this study, we further used GENIE3 to evaluate the top 50 TFs as the candidates. Combining WGCNA and GENIE3 tow algorithms, five crucial TFs (*OsCBF3*, *OsZHD8*, *OsTZF1*, *OsCSA*, and *OsIG1*) associated with the low-temperature resistance in rice were predicted. *OsCBF3*/*DREB1A* has been proven to bind with multiple genes and TFs to regulate the cold resistance of downstream genes (Jia et al., 2022). *OsZHD8* can bind to the promoter of *OsDREB1B*, which is specifically induced by low temperature, and shows an increasing expression after ABA treatment in rice (Figueiredo et al., 2012), suggesting that it might have function on the chilling stress. Overexpression of *OsTZF1* causes ABA-induced growth inhibition of rice seedlings compared with WT, suggesting *OsTZF1* positively regulates ABA response (Zhang et al., 2012). Meanwhile, the high expression level of *OsTZF1* enhances resistance to hydrogen peroxide, salt, and drought stresses, suggesting that *OsTZF1* might be a multiple abiotic stress resistant regulators and probably play an important role in resisting chilling stress (Jan et al., 2013). The R2R3 MYB TF *OsCSA* can increase adaptations to adverse environments by balancing glucose and ABA homeostasis (Sun et al., 2021). *OsIG1* plays an important role in regulating rice floral organs development (Zhang et al., 2015), and its role in cold stress and ABA signaling pathways is currently unclear. Altogether, our results suggest the combination WGCNA and GENIE3 would be an efficient way to predict hub/key regulators in complicated networks.

In the original article, combining analysis of the differentially accumulated metabolites (DAMs) and DEGs, it has been predicted that galactinol, b-alanine, glutamate, naringenin, serotonin, ABA, and *OsNCED3* might be involved in the chilling tolerance variation of 9311 and DC90 (Cen et al., 2020). However, core TFs involved in regulating the biological processes of chilling stress remain unclear. In our study, crucial genes/TFs of low temperature tolerance were identified through WGCNA in combination with GENIE3 network based on DEGs. In addition, we identified a lot of TFs and constructed a potential TF regulatory network. Our results not only reveal common candidates (such as the ABA pathway, and *OsNCED3*), but also uncover more crucial TFs, which may reflect the application of different prediction methods. These two sets of results could be complemented to each other and will provide more candidate genes for rice breeding and deepen our understanding mechanisms underlying how the rice resists low temperature.

## 5 Conclusions

In this study, we performed a transcriptome analysis of rice seedlings in two cultivars under chilling stress at 6 time points. We identified 3,590 DEGs including chilling-responsive genes that were dynamically regulated under low temperature. Specifically, abscisic acid biosynthesis and signaling, coupled with transcription regulator activity, emerges as closely intertwined with the low-temperature resistance of rice. The integration analysis of hub gene networks has shed light on the key TFs triggered in response to chilling conditions. Through the correlation investigation of WGCNA and GRN, five TF candidates were predicted involved in the processes of chilling response. Our results provide invaluable gene resources for breeding climate resilient rice.

## Data availability statement

The original contributions presented in the study are included in the article/**Supplementary Material**. Further inquiries can be directed to the corresponding author.

## Author contributions

RZ: Conceptualization, Formal analysis, Methodology, Writing – original draft. XX: Formal analysis, Investigation, Writing – review & editing. XC: Writing – review & editing, Investigation. YW: Investigation, Writing – review & editing. MZ: Conceptualization, Funding acquisition, Methodology, Writing – review & editing.

## Supplementary material

Supplementary Figure 1 GO enrichment for different modules selected before. Including **(A)** dark green, **(B)** turquoise, **(C)** magenta, **(D)** dark red, **(E)** dark gray, and **(F)** dark orange modules.

Supplementary Figure 2 Heatmap showing the expression patterns of DEGs related to BR pathways between DC90 and 9311 under 72h chilling treatment and 24h recovery. The color gradient indicates the normalized FPKM value (z-score) of genes [high expression (red) and low expression (blue)].

Supplementary Figure 3 Heatmap showing the expression patterns of DEGs related to CTK pathways between DC90 and 9311 under 72h chilling treatment and 24h recovery. The color gradient indicates the normalized FPKM value (z-score) of genes [high expression (red) and low expression (blue)].

Supplementary Figure 4 Heatmap showing the expression patterns of DEGs related to Eth pathways between DC90 and 9311 under 72h chilling treatment and 24h recovery. The color gradient indicates the normalized FPKM value (z-score) of genes [high expression (red) and low expression (blue)].

Supplementary Figure 5 Heatmap showing the expression patterns of DEGs related to GA pathways between DC90 and 9311 under 72h chilling treatment and 24h recovery. The color gradient indicates the normalized FPKM value (z-score) of genes [high expression (red) and low expression (blue)].

Supplementary Figure 6 Heatmap showing the expression patterns of DEGs related to IAA pathways between DC90 and 9311 under 72h chilling treatment and 24h recovery. The color gradient indicates the normalized FPKM value (z-score) of genes [high expression (red) and low expression (blue)].

Supplementary Figure 7 Heatmap showing the expression patterns of DEGs related to JA pathways between DC90 and 9311 under 72h chilling treatment and 24h recovery. The color gradient indicates the normalized FPKM value (z-score) of genes [high expression (red) and low expression (blue)].

## References

[B1] Bolger A. M. Lohse M. Usadel B. (2014). Trimmomatic: a flexible trimmer for Illumina sequence data. Bioinformatics 30, 2114–2120. doi: 10.1093/bioinformatics/btu170 24695404 PMC4103590

[B2] Cao S. F. Cai Y. T. Yang Z. F. Zheng Y. H. (2012). MeJA induces chilling tolerance in loquat fruit by regulating proline and γ-aminobutyric acid contents. Food Chem. 133, 1466–1470. doi: 10.1016/j.foodchem.2012.02.035

[B3] Cen W. Liu J. Lu S. Jia P. Yu K. Han Y.. (2018). Comparative proteomic analysis of QTL CTS-12 derived from wild rice (Oryza rufipogon Griff.), in the regulation of cold acclimation and de-acclimation of rice (*Oryza sativa* L.) in response to severe chilling stress. BMC Plant Biol. 18, 163. doi: 10.1186/s12870-018-1381-7 30097068 PMC6086036

[B4] Cen W. J. Zhao W. L. Ma M. Q. Lu S. Y. Liu J. B. Cao Y. Q.. (2020). The wild rice locus mediates ABA-dependent stomatal opening modulation to limit water loss under severe chilling stress. Front. Plant Sci. 11. doi: 10.3389/fpls.2020.575699 PMC7661758 33193516

[B5] Chen J. Zhang L. Liu Y. Shen X. Guo Y. Ma X.. (2024). RNA-Seq-based WGCNA and association analysis reveal the key regulatory module and genes responding to salt stress in wheat roots. Plants 13, 274. doi: 10.3390/plants13020274 38256827 PMC10818790

[B6] Chinnusamy V. Zhu J. Zhu J. K. (2006). Gene regulation during cold acclimation in plants. Physiol. Plant 126, 52–61. doi: 10.1111/j.1399-3054.2006.00596.x

[B7] Conway J. R. Lex A. Gehlenborg N. (2017). UpSetR: an R package for the visualization of intersecting sets and their properties. Bioinformatics 33, 2938–2940. doi: 10.1093/bioinformatics/btx364 28645171 PMC5870712

[B8] Cui Z. Xue C. Mei Q. Xuan Y. (2022). Malectin domain protein Kinase (MDPK) promotes rice resistance to sheath blight via IDD12, IDD13, and IDD14. Int. J. Mol. Sci. 23, 8214. doi: 10.3390/ijms23158214 35897795 PMC9331740

[B9] Ding Y. Shi Y. Yang S. (2019). Advances and challenges in uncovering cold tolerance regulatory mechanisms in plants. New Phytol. 222, 1690–1704. doi: 10.1111/nph.15696 30664232

[B10] Figueiredo D. D. Barros P. M. Cordeiro A. M. Serra T. S. Lourenco T. Chander S.. (2012). Seven zinc-finger transcription factors are novel regulators of the stress responsive gene OsDREB1B. J. Exp. Bot. 63, 3643–3656. doi: 10.1093/jxb/ers035 22412187

[B11] Ginestet C. (2011). ggplot2: Elegant graphics for data analysis. J. R. Stat. Soc A. 174, 245–245. doi: 10.1111/j.1467-985X.2010.00676_9.x

[B12] Gnanaraj J. (2008). Minimally invasive appendicectomy using the cytoscope. Trop. Doct. 38, 14–15. doi: 10.1258/td.2007.060125 18302852

[B13] Guan Y. L. Hwarari D. Korboe H. M. Ahmad B. Cao Y. W. Movahedi A.. (2023). Low temperature stress-induced perception and molecular signaling pathways in plants. Environ. Exp. Bot. 207, 105190. doi: 10.1016/j.envexpbot.2022.105190

[B14] He R. Y. Wang X. S. Wang G. J. (1990). Effects of brassinolide (Br) on the growth and chilling resistance of maize. Abstr. Pap. Am. Chem. S. 200, 220–230. doi: 10.1021/bk-1991-0474.ch019

[B15] Hu Y. Ma M. Zhao W. Niu P. Li R. Luo J. (2024). Identification of hub genes involved in GA-regulated coleoptile elongation under submerged germinations in rice. J. Exp. Bot. 75, 3862–3876. doi: 10.1093/jxb/erae144 38571323

[B16] Hubbard T. Barker D. Birney E. Cameron G. Chen Y. Clark L.. (2002). The Ensembl genome database project. Nucleic Acids Res. 30, 38–41. doi: 10.1093/nar/30.1.38 11752248 PMC99161

[B17] Jan A. Maruyama K. Todaka D. Kidokoro S. Abo M. Yoshimura E.. (2013). OsTZF1, a CCCH-tandem zinc finger protein, confers delayed senescence and stress tolerance in rice by regulating stress-related genes. Plant Physiol. 161, 1202–1216. doi: 10.1104/pp.112.205385 23296688 PMC3585590

[B18] Jia M. Meng X. Song X. Zhang D. Kou L. Zhang J.. (2022). Chilling-induced phosphorylation of IPA1 by OsSAPK6 activates chilling tolerance responses in rice. Cell Discovery 8, 71. doi: 10.1038/s41421-022-00413-2 35882853 PMC9325753

[B19] Kaufmann K. Pajoro A. Angenent G. C. (2010). Regulation of transcription in plants: mechanisms controlling developmental switches. Nat. Rev. Genet. 11, 830–842. doi: 10.1038/nrg2885 21063441

[B20] Kim D. Paggi J. M. Park C. Bennett C. Salzberg S. L. (2019). Graph-based genome alignment and genotyping with HISAT2 and HISAT-genotype. Nat. Biotechnol. 37, 907–915. doi: 10.1038/s41587-019-0201-4 31375807 PMC7605509

[B21] Kuang J. F. Wu C. J. Guo Y. F. Walther D. Shan W. Chen J. Y.. (2021). Deciphering transcriptional regulators of banana fruit ripening by regulatory network analysis. Plant Biotechnol. J. 19, 477–489. doi: 10.1111/pbi.13477 32920977 PMC7955892

[B22] Kumar S. Shah S. H. Vimala Y. Jatav H. S. Ahmad P. Chen Y. L.. (2022). Abscisic acid: metabolism, transport, crosstalk with other plant growth regulators, and its role in heavy metal stress mitigation. Front. Plant Sci. 13. doi: 10.3389/fpls.2022.972856 PMC9515544 36186053

[B23] Langfelder P. Horvath S. (2008). WGCNA: an R package for weighted correlation network analysis. BMC Bioinf. 9, 1–13. doi: 10.1186/1471-2105-9-559 PMC2631488 19114008

[B24] Li M. Duan X. Gao G. Liu T. Qi H. (2022). Running title: ABA pathway meets CBF pathway at CmADC. Hortic. Res. 9, uhac002. doi: 10.1093/hr/uhac002 35147169 PMC9016860

[B25] Li M. Lin L. Zhang Y. Sui N. (2019). ZmMYB31, a R2R3-MYB transcription factor in maize, positively regulates the expression of CBF genes and enhances resistance to chilling and oxidative stress. Mol. Biol. Res. 46, 3937–3944. doi: 10.1007/s11033-019-04840-5 31037550

[B26] Li R. Song Y. Wang X. Zheng C. Liu B. Zhang H.. (2024). OsNAC5 orchestrates OsABI5 to fine-tune cold tolerance in rice. J. Integr. Plant Biol. 66, 660–682. doi: 10.1111/jipb.13585 37968901

[B27] Liao Y. Smyth G. K. Shi W. (2014). featureCounts: an efficient general purpose program for assigning sequence reads to genomic features. Bioinformatics 30, 923–930. doi: 10.1093/bioinformatics/btt656 24227677

[B28] Liu J. Liu J. He M. Zhang C. Liu Y. Li X.. (2023). OsMAPK6 positively regulates rice cold tolerance at seedling stage via phosphorylating and stabilizing OsICE1 and OsIPA1. TAG. Theor. Appl. Genet. 137, 10. doi: 10.1007/s00122-023-04506-8 38103049

[B29] Love M. I. Huber W. Anders S. (2014). Moderated estimation of fold change and dispersion for RNA-seq data with DESeq2. Genome Biol. 15, 1–21. doi: 10.1186/s13059-014-0550-8 PMC4302049 25516281

[B30] Lukatkin A. S. (2005). Initiation and development of chilling injury in leaves of chilling-sensitive plants. Russ. J. Plant Physl. 52, 542–546. doi: 10.1007/s11183-005-0080-z

[B31] Lukatkin A. S. Brazaityte A. Bobinas C. Duchovskis P. (2012). Chilling injury in chilling-sensitive plants: a review. Zemdirbyste 99, 111–124. doi: 10.21273/HORTSCI.21.6.1329

[B32] Mao D. Chen C. (2012). Colinearity and similar expression pattern of rice DREB1s reveal their functional conservation in the cold-responsive pathway. PLoS One 7, e47275. doi: 10.1371/journal.pone.0047275 23077584 PMC3473061

[B33] Nawaz M. Sun J. F. Shabbir S. Khattak W. A. Ren G. Q. Nie X. J.. (2023). A review of plants strategies to resist biotic and abiotic environmental stressors. Sci. Total Environ. 900, 165832. doi: 10.1016/j.scitotenv.2023.165832 37524179

[B34] Nie Y. Guo L. Cui F. Shen Y. Ye X. Deng D.. (2022). Innovations and stepwise evolution of CBFs/DREB1s and their regulatory networks in angiosperms. J. Integr. Plant Biol. 64, 2111–2125. doi: 10.1111/jipb.13357 36070250

[B35] Seki M. Narusaka M. Abe H. Kasuga M. Yamaguchi-Shinozaki K. Carninci P.. (2001). Monitoring the expression pattern of 1300 Arabidopsis genes under drought and cold stresses by using a full-length cDNA microarray. Plant Cell 13, 61–72. doi: 10.1105/tpc.13.1.61 11158529 PMC102214

[B36] Shafi S. Shafi I. Zaffar A. Zargar S. M. Shikari A. B. Ranjan A.. (2023). The resilience of rice under water stress will be driven by better roots: Evidence from root phenotyping, physiological, and yield experiments. Plant Stress 10, 100211. doi: 10.1016/j.stress.2023.100211

[B37] Shu P. Li Y. J. Xiang L. T. Sheng J. P. Shen L. (2022). Ethylene enhances tolerance to chilling stress in tomato fruit partially through the synergistic regulation between antioxidant enzymes and ATP synthases. Postharvest Biol. Tec. 193, 112065. doi: 10.1016/j.postharvbio.2022.112065

[B38] Song Z. Lai X. Chen H. Wang L. Pang X. Hao Y.. (2022). Role of MaABI5-like in abscisic acid-induced cold tolerance of 'Fenjiao' banana fruit. Hortic. Res. 9, uhac130. doi: 10.1093/hr/uhac130 36936195 PMC10021067

[B39] Sun J. Zheng T. H. Yu J. Wu T. T. Wang X. H. Chen G. M.. (2017). TSV, a putative plastidic oxidoreductase, protects rice chloroplasts from cold stress during development by interacting with plastidic thioredoxin Z. New Phytol. 215, 240–255. doi: 10.1111/nph.14482 28248438

[B40] Sun L. Yuan Z. Wang D. Li J. Shi J. Hu Y.. (2021). Carbon starved anther modulates sugar and ABA metabolism to protect rice seed germination and seedling fitness. Plant Physiol. 187, 2405–2418. doi: 10.1093/plphys/kiab391 34618084 PMC8644061

[B41] Thomashow M. F. (1999). Plant cold acclimation: Freezing tolerance genes and regulatory mechanisms. Annu. Rev. Plant Biol. 50, 571–599. doi: 10.1146/annurev.arplant.50.1.571 15012220

[B42] Ueda Y. Ohtsuki N. Kadota K. Tezuka A. Nagano A. J. Kadowaki T.. (2020). Gene regulatory network and its constituent transcription factors that control nitrogen-deficiency responses in rice. New Phytol. 227, 1434–1452. doi: 10.1111/nph.16627 32343414

[B43] Vân A. H. T. Geurts P. (2018). dynGENIE3: dynamical GENIE3 for the inference of gene networks from time series expression data. Sci. Rep. 8, 3384. doi: 10.1038/s41598-018-21715-0 29467401 PMC5821733

[B44] Wu T. Z. Hu E. Q. Xu S. B. Chen M. J. Guo P. F. Dai Z. H.. (2021). clusterProfiler 4.0: A universal enrichment tool for interpreting omics data. Innovation-Amsterdam 2, 100141. doi: 10.1016/j.xinn.2021.100141 PMC8454663 34557778

[B45] Xiang H. T. Wang T. T. Zheng D. F. Wang L. Z. Feng Y. J. Luo Y.. (2017). ABA pretreatment enhances the chilling tolerance of a chilling-sensitive rice cultivar. Braz. J. Bot. 40, 853–860. doi: 10.1007/s40415-017-0409-9

[B46] Xie G. Zou X. Liang Z. Zhang K. Wu D. Jin H.. (2024). GBF family member PfGBF3 and NAC family member PfNAC2 regulate rosmarinic acid biosynthesis under high light. Plant Physiol. 195, 1728–1744. doi: 10.1093/plphys/kiae036 38441888

[B47] Xu L. M. Zhou L. Zeng Y. W. Wang F. M. Zhang H. L. Shen S. Q.. (2008). Identification and mapping of quantitative trait loci for cold tolerance at the booting stage in a rice near-isogenic line. Plant Sci. 174, 340–347. doi: 10.1016/j.plantsci.2007.12.003

[B48] Yin Q. Qin W. Zhou Z. Wu A. M. Deng W. Li Z.. (2024). Banana MaNAC1 activates secondary cell wall cellulose biosynthesis to enhance chilling resistance in fruit. Plant Biol. J. 22, 413–426. doi: 10.1111/pbi.14195 PMC10826994 37816143

[B49] Zhang J. R. Tang W. Huang Y. L. Niu X. L. Zhao Y. Han Y.. (2015). Down-regulation of a LBD-like gene, OsIG1, leads to occurrence of unusual double ovules and developmental abnormalities of various floral organs and megagametophyte in rice. J. Exp. Bot. 66, 99–112. doi: 10.1093/jxb/eru396 25324400 PMC4265153

[B50] Zhang C. Zhang F. Zhou J. Fan Z. Chen F. Ma H.. (2012). Overexpression of a phytochrome-regulated tandem zinc finger protein gene, OsTZF1, confers hypersensitivity to ABA and hyposensitivity to red light and far-red light in rice seedlings. Plant Cell Rep. 31, 1333–1343. doi: 10.1007/s00299-012-1252-x 22572927

[B51] Zhang M. Zhao R. Huang K. Huang S. Wang H. Wei Z.. (2022). The OsWRKY63-OsWRKY76-OsDREB1B module regulates chilling tolerance in rice. Plant J. 112, 383–398. doi: 10.1111/tpj.15950 35996876

[B52] Zhu N. Cheng S. Liu X. Du H. Dai M. Zhou D. X.. (2015). The R2R3-type MYB gene OsMYB91 has a function in coordinating plant growth and salt stress tolerance in rice. Plant Sci. 236, 146–156. doi: 10.1016/j.plantsci.2015.03.023 26025528

