# Supplementary figures and images for: Comparing time-series transcriptomes between chilling-resistant and -susceptible rice reveals potential transcription factors responding to chilling stress
Source: Front Plant Sci. 2024 Aug 6;15:1451403. doi: 10.3389/fpls.2024.1451403 (PMC11333254; doi:10.3389/fpls.2024.1451403)

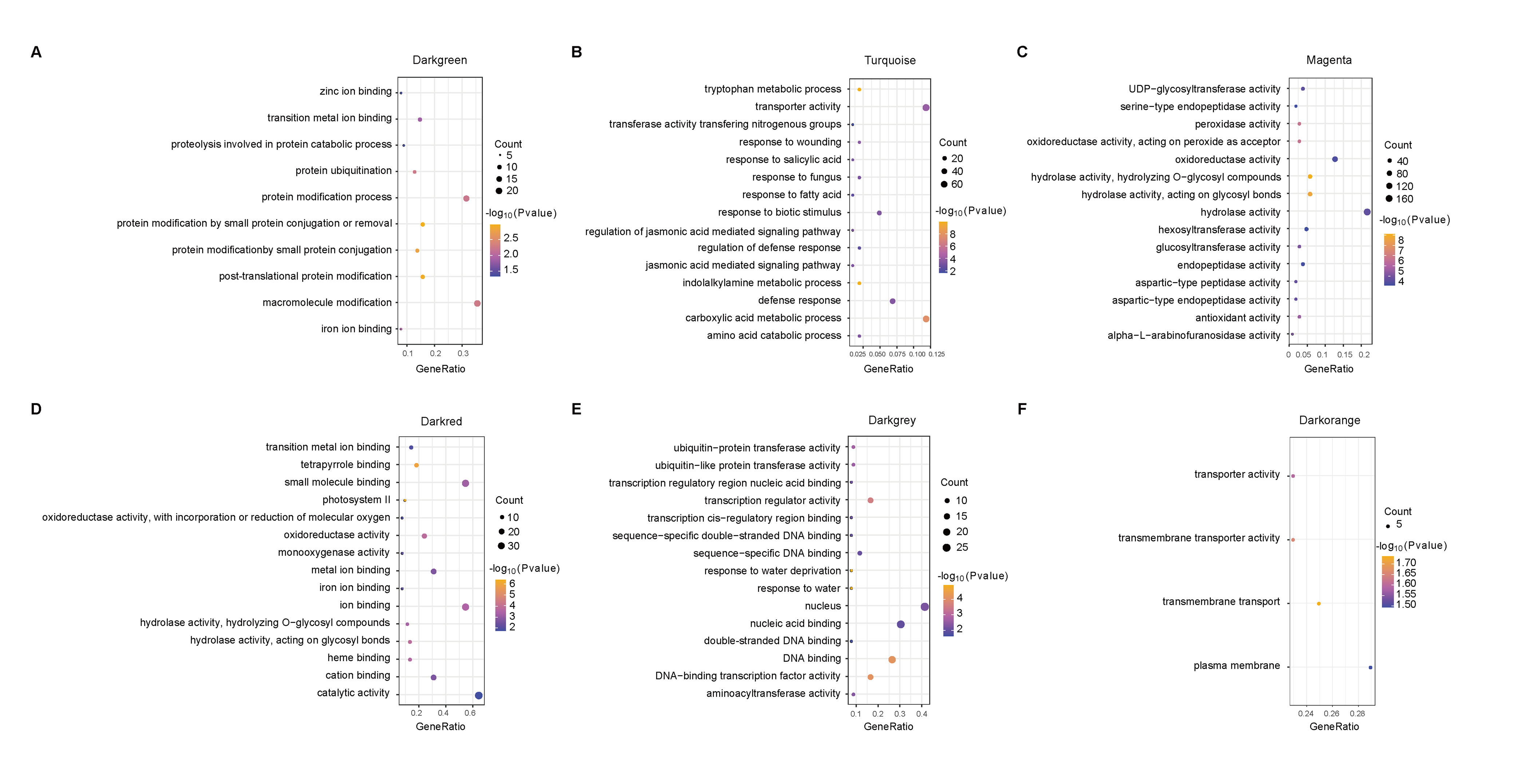

Supplement: Supplementary Figure 1 — GO enrichment for different modules selected before. Including (A) dark green, (B) turquoise, (C) magenta, (D) dark red, (E) dark gray, and (F) dark orange modules. [file Image_1.tif]

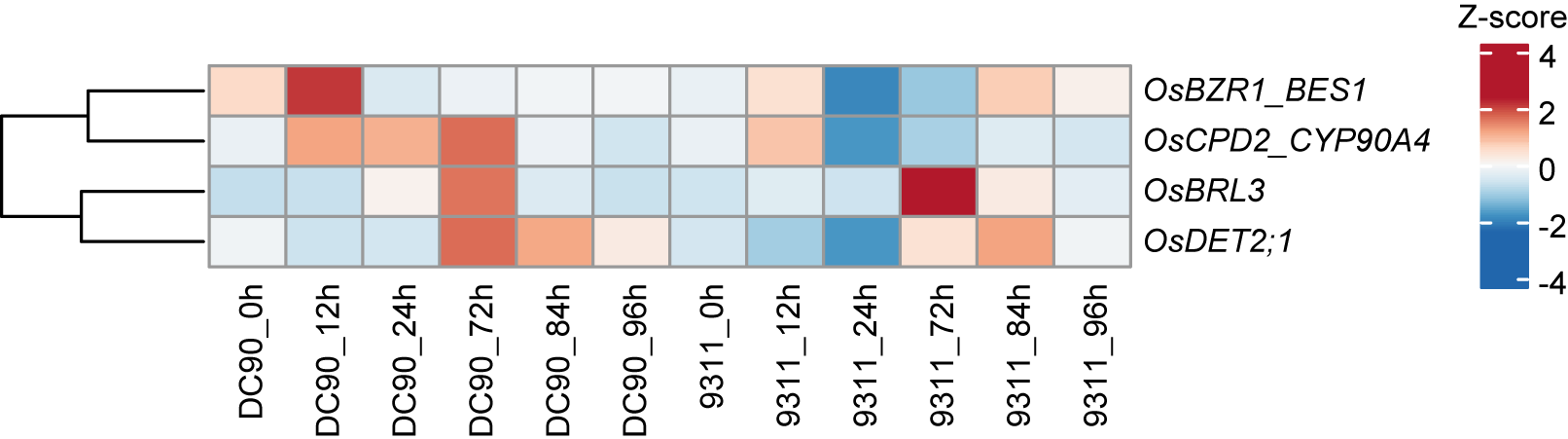

Supplement: Supplementary Figure 2 — Heatmap showing the expression patterns of DEGs related to BR pathways between DC90 and 9311 under 72h chilling treatment and 24h recovery. The color gradient indicates the normalized FPKM value (z-score) of genes [high expression (red) and low expression (blue)]. [file Image_2.tif]

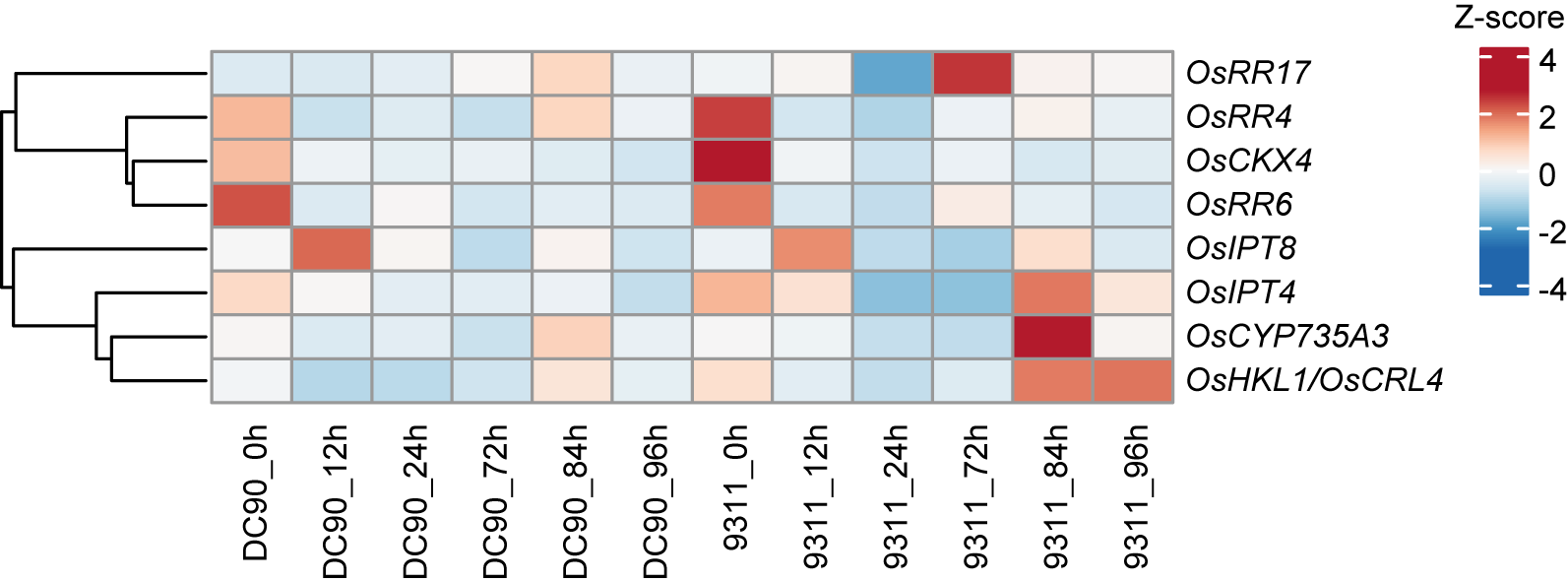

Supplement: Supplementary Figure 3 — Heatmap showing the expression patterns of DEGs related to CTK pathways between DC90 and 9311 under 72h chilling treatment and 24h recovery. The color gradient indicates the normalized FPKM value (z-score) of genes [high expression (red) and low expression (blue)]. [file Image_3.tif]

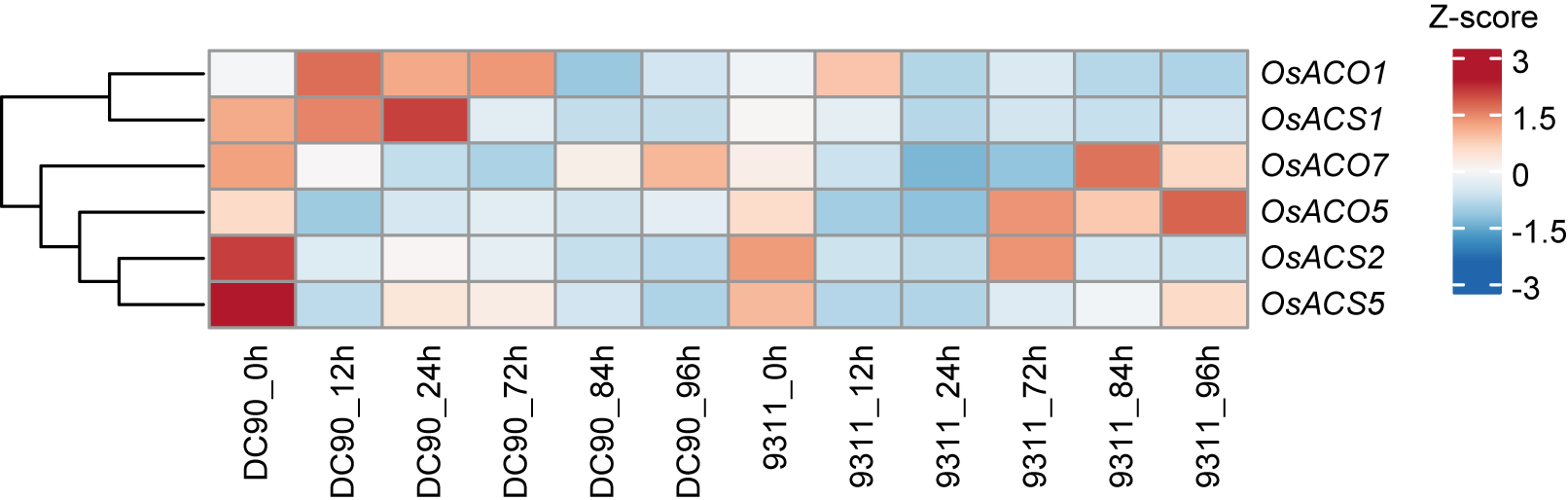

Supplement: Supplementary Figure 4 — Heatmap showing the expression patterns of DEGs related to Eth pathways between DC90 and 9311 under 72h chilling treatment and 24h recovery. The color gradient indicates the normalized FPKM value (z-score) of genes [high expression (red) and low expression (blue)]. [file Image_4.tif]

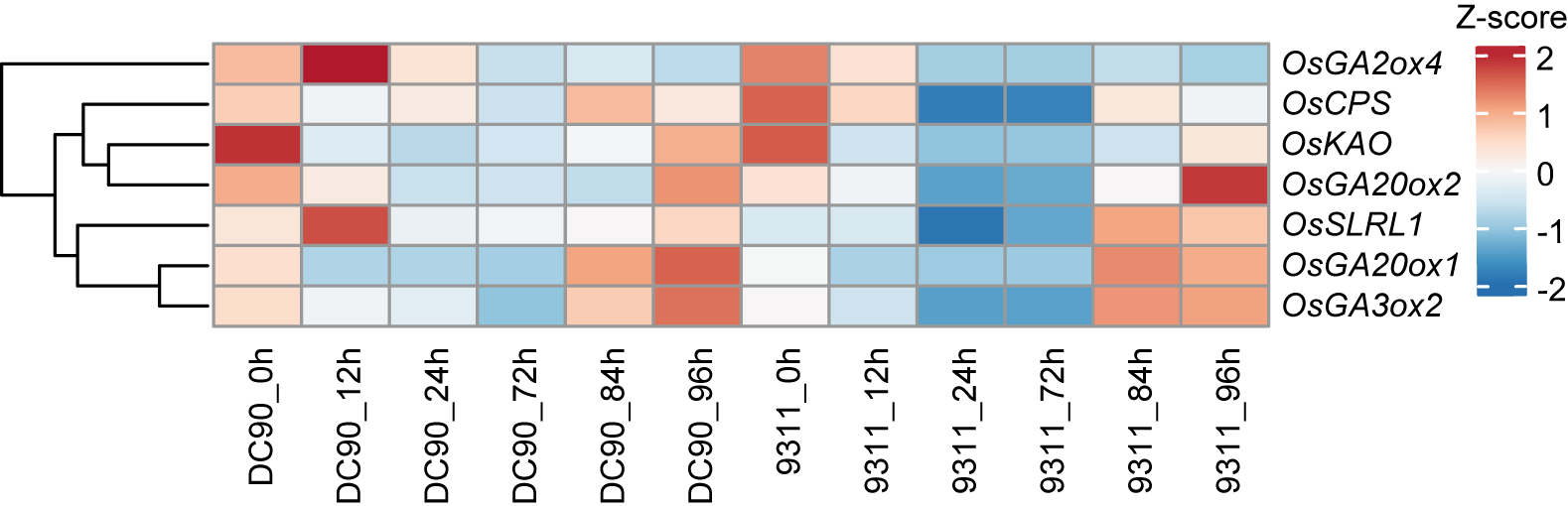

Supplement: Supplementary Figure 5 — Heatmap showing the expression patterns of DEGs related to GA pathways between DC90 and 9311 under 72h chilling treatment and 24h recovery. The color gradient indicates the normalized FPKM value (z-score) of genes [high expression (red) and low expression (blue)]. [file Image_5.tif]

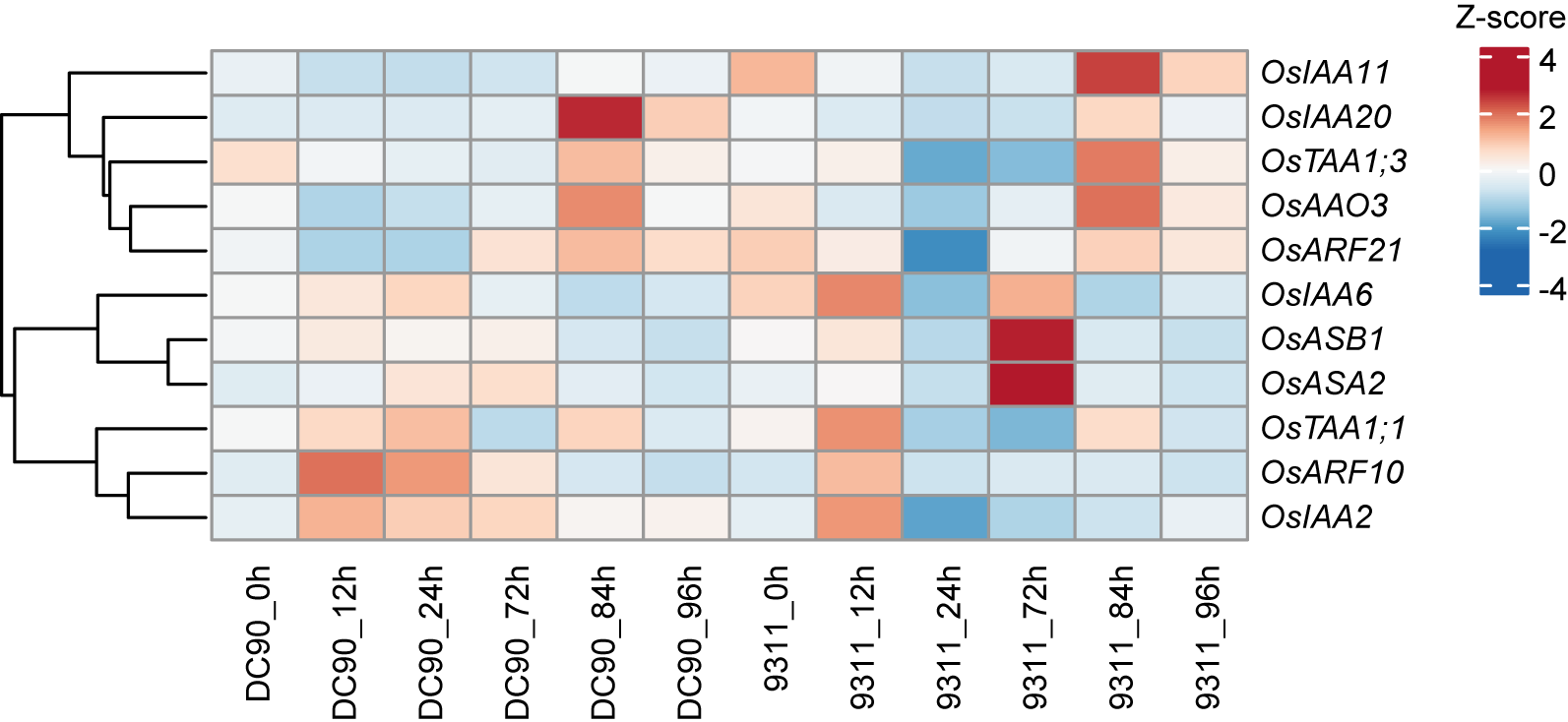

Supplement: Supplementary Figure 6 — Heatmap showing the expression patterns of DEGs related to IAA pathways between DC90 and 9311 under 72h chilling treatment and 24h recovery. The color gradient indicates the normalized FPKM value (z-score) of genes [high expression (red) and low expression (blue)]. [file Image_6.tif]

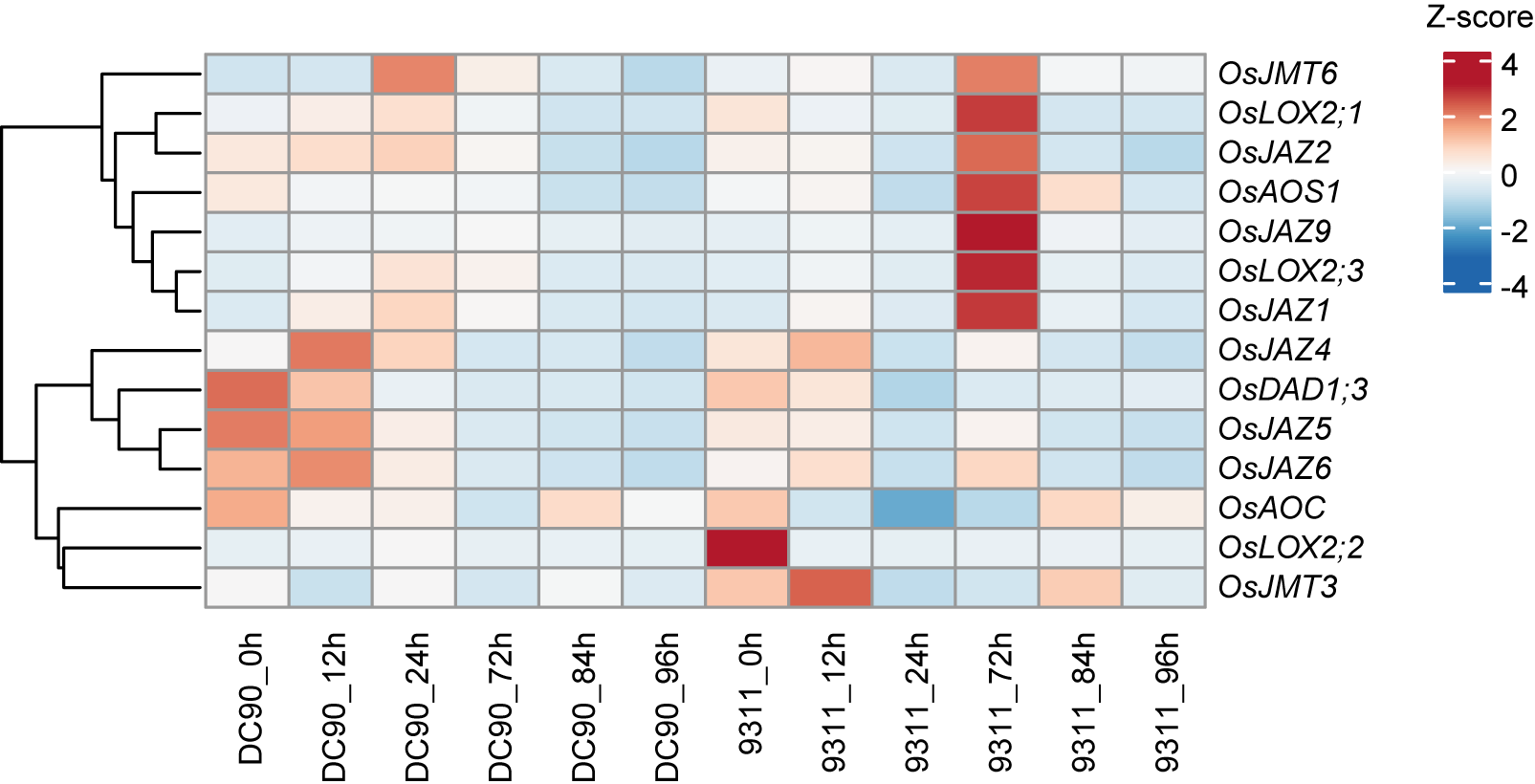

Supplement: Supplementary Figure 7 — Heatmap showing the expression patterns of DEGs related to JA pathways between DC90 and 9311 under 72h chilling treatment and 24h recovery. The color gradient indicates the normalized FPKM value (z-score) of genes [high expression (red) and low expression (blue)]. [file Image_7.tif]
